# Prescription and Dispensation of QT-Prolonging Medications in Individuals Receiving Hemodialysis

**DOI:** 10.1001/jamanetworkopen.2024.8732

**Published:** 2024-04-30

**Authors:** Virginia Wang, Chin-Hua (Lily) Wang, Magdalene M. Assimon, Patrick H. Pun, Wolfgang C. Winkelmayer, Jennifer E. Flythe

**Affiliations:** 1 Department of Population Health Sciences, Duke University School of Medicine, Durham, North Carolina; 2 Center of Innovation to Accelerate Discovery and Practice Transformation, Durham Veterans Affairs Medical Center, Durham, North Carolina; 3 Department of Medicine, Duke University School of Medicine, Durham, North Carolina; 4 The Cecil G. Sheps Center for Health Services Research, The University of North Carolina at Chapel Hill, Chapel Hill; 5 Aetion, Inc, New York, New York; 6 Duke Clinical Research Institute, Duke University Medical Center, Durham, North Carolina; 7 Selzman Institute for Kidney Health, Section of Nephrology, Baylor College of Medicine, Houston, Texas; 8 University of North Carolina Kidney Center, Division of Nephrology and Hypertension, Department of Medicine, The University of North Carolina School of Medicine, Chapel Hill

## Abstract

**Importance:**

Individuals with dialysis-dependent kidney failure have numerous risk factors for medication-related adverse events, including receipt of care by multiple clinicians and initiation of some QT-prolonging medications with known risk of torsades de pointes (TdP), which is associated with higher risk of sudden cardiac death. Little is known about the prescription and dispensation patterns of QT-prolonging medications among people receiving dialysis, hindering efforts to reduce drug-related harm from these and other medications in this high-risk population.

**Objective:**

To examine prescription and dispensation patterns of QT-prolonging medications with known TdP risk and selected interacting medications prescribed to individuals receiving hemodialysis.

**Design, Setting, and Participants:**

This cross-sectional study included patients 60 years or older who were enrolled in Medicare Parts A, B, and D receiving in-center hemodialysis from January 1 to December 31, 2019. Analyses were conducted from October 20, 2022, to June 16, 2023.

**Exposures:**

New-user prescriptions for the 7 most frequently filled QT-prolonging medications characterized by the timing of the new prescription relative to acute care encounters, the type of prescribing clinician and pharmacy that dispensed the medication, and concomitant use of selected medications known to interact with the 7 most frequently filled QT-prolonging medications with known TdP risk.

**Main Outcomes and Measures:**

The main outcomes were the frequencies of the most commonly filled and new-use episodes of QT-prolonging medications; the timing of medication fills relative to acute care events; prescribers and dispensing pharmacy characteristics for new use of medications; and the frequency and types of new-use episodes with concurrent use of potentially interacting medications.

**Results:**

Of 20 761 individuals receiving hemodialysis in 2019 (mean [SD] age, 74 [7] years; 51.1% male), 10 992 (52.9%) filled a study drug prescription. Approximately 80% (from 78.6% for odansetron to 93.9% for escitalopram) of study drug new-use prescriptions occurred outside of an acute care event. Between 36.8% and 61.0% of individual prescriptions originated from general medicine clinicians. Between 16.4% and 26.2% of these prescriptions occurred with the use of another QT-prolonging medication. Most potentially interacting drugs were prescribed by different clinicians (46.3%-65.5%).

**Conclusions and Relevance:**

In this cross-sectional study, QT-prolonging medications for individuals with dialysis-dependent kidney failure were commonly prescribed by nonnephrology clinicians and from nonacute settings. Prescriptions for potentially interacting medications often originated from different prescribers. Strategies aimed at minimizing high-risk medication-prescribing practices in the population undergoing dialysis are needed.

## Key Points

**Question:**

What are the prescription and dispensation patterns of QT-prolonging medications with known torsades de pointes risk and selected interacting medications for people receiving hemodialysis?

**Findings:**

This cross-sectional study of 20 761 adults 60 years or older found that QT-prolonging medications for individuals with dialysis-dependent kidney failure were commonly prescribed by nonnephrology clinicians and from nonacute settings. Prescriptions for potentially interacting medications often originated from different prescribers.

**Meaning:**

The findings of this study suggest that clinician- and health system–level strategies aimed at minimizing high-risk medication–prescribing practices in the population undergoing dialysis are needed.

## Teaser

This cross-sectional study of older adults with kidney failure receiving hemodialysis examines prescription and dispensation patterns of QT-prolonging medications with known torsades de pointes risk and selected interacting medications prescribed to this population.

## Introduction

There are approximately 500 000 people with hemodialysis-dependent kidney failure in the US,^1^ and these individuals have a tremendous burden of comorbid disease that often requires care by multiple clinicians and treatment with numerous medications. On average, patients receiving hemodialysis are cared for simultaneously by clinicians from 5 different specialties^2,3^ and are prescribed 10 to 12 daily medications.^4,5^ Evidence from other chronic disease populations shows that individuals with more (vs fewer) medication prescribers have higher risks of drug interactions,^6,7^ receipt of inappropriate medications,^8^ and occurrence of adverse drug events.^9^ Findings are similar among patients who receive prescriptions from acute (vs ambulatory) care settings and/or clinicians not involved in their long-term care.^8^

Individuals with dialysis-dependent kidney failure may be particularly vulnerable to medication-related harms, as the impacts of kidney dysfunction on drug pharmacokinetics (ie, absorption, distribution, metabolism, and excretion)^10^ and pharmacodynamics (ie, the physiologic drug effects)^11^ can increase the risk of drug-related adverse events. For example, compared with clinically relevant comparator medications, QT-prolonging medications with known risks of torsades de pointes (TdP), such as respiratory fluoroquinolones, azithromycin, ondansetron, and certain selective serotonin reuptake inhibitors, are associated with higher risks of sudden cardiac death in people receiving hemodialysis.^12,13,14,15^ This risk is further elevated in the setting of concomitant use of other QT-prolonging medications with known risk of TdP than in the setting of stand-alone use.^12,14^ Prescriber education programs,^16,17^ medication reconciliation,^18,19,20^ and electronic health record (EHR) supports^21,22,23^ reduce unsafe prescribing practices in populations not undergoing dialysis. However, the absence of data on the origins of prescriptions for high-risk medications among people receiving dialysis hinders targeted implementation of such risk-mitigation strategies in the population undergoing dialysis.

To address this knowledge gap, we undertook this study to investigate the origins of prescriptions for QT-prolonging medications with known risks of TdP. We also selected interacting medications in a sample of older individuals with hemodialysis-dependent kidney failure in the Medicare fee-for-service (FFS) and Part D programs.

## Methods

### Study Design, Data Source, and Cohort

The University of North Carolina at Chapel Hill Institutional Review Board reviewed this study. A waiver of the requirement to obtain informed consent was granted because of the study’s large size, data anonymity, and retrospective nature. We followed the Strengthening the Reporting of Observational Studies in Epidemiology (STROBE) reporting guideline for cross-sectional studies.^24^

We conducted a cross-sectional study using the Medicare Master Beneficiary Summary File, as well as institutional, clinician, and prescription drug claims (Parts A, B, and D, respectively) from a random 20% sample of Medicare FFS beneficiaries 60 years or older (ages available in data source) to identify individuals receiving in-center hemodialysis as of January 1 (the study index date) through December 31, 2019. For cohort inclusion, we required that individuals have a diagnosis code for end-stage kidney disease (*International Statistical Classification of Diseases and Related Health Problems, Tenth Revision* code N18.6) and 1 or more claims for outpatient dialysis treatment with Medicare as the primary payer in the 6-month baseline period (July 1 through December 31, 2018) (eTable 1 in Supplement 1). We excluded beneficiaries who received home dialysis or hospice care, underwent a kidney transplant, died, enrolled in Medicare Advantage, or did not have continuous Medicare Part A, B, or D coverage either during the baseline period or on January 1, 2019. After the index date, we stopped observing individuals’ medication use at the onset of any of the exclusionary events listed above.

### QT-Prolonging Medication Use

We used the CredibleMeds website to compile a list of medications that can cause QT prolongation and/or TdP (eTable 2 in Supplement 1).^25^ CredibleMeds is an online resource that relies on up-to-date information from published literature, medication package inserts, the US Food and Drug Administration’s Adverse Event Reporting System, and other sources to classify QT-prolonging medications as having a known, possible, or conditional risk of TdP.^25^ We used Medicare Part D claims to ascertain each beneficiary’s outpatient use of a QT-prolonging medication with known TdP risk during 2019.

We then created separate new-user cohorts of the 7 most frequently filled QT-prolonging medications with known TdP risk in 2019 (hereinafter described as the 7 new-user cohorts): azithromycin, ondansetron, levofloxacin, ciprofloxacin, amiodarone, escitalopram, and fluconazole (eTable 3 in Supplement 1). The focus on a limited number of high-risk medications allowed for more detailed, medication-specific descriptions of prescription and dispensation patterns of medications shown to be associated with a higher risk of sudden cardiac death among people receiving maintenance hemodialysis.^12,13,14,15^ We defined new use as a prescription fill for a study medication after a 180-day washout period free of prescription fills for the study drug of interest. Studying new use supported reliable ascertainment of the origin of study prescription medications (ie, prescriber and dispenser) and captured the point in time when the prescriber made the prescribing decision and might have considered medications that were already prescribed to the patient in their decision to prescribe the study medication.

### Cohort Characterization

Covariates were ascertained in the baseline period using the Medicare Master Beneficiary Summary File and claims data and included demographics, comorbid conditions, concomitant medication use, and health care utilization metrics (eTable 1 in Supplement 1). Race and ethnicity categories included Black, Hispanic, non-Hispanic White, and other (Asian or Pacific Islander, American Indian or Alaska Native, other, and unknown). Race and ethnicity were ascertained by Medicare enrollment data and were included in the study to characterize cohort demographics. Groups in the other category were collapsed due to small individual sample sizes. Comorbid conditions were considered present if there was an applicable diagnosis code located in any position on 1 or more inpatient claims or on 2 or more outpatient claims during the 180-day baseline period. Concomitant medication use was determined on January 1, 2019, using Part D prescription drug claims. Polypharmacy was defined as use of 5 or more prescribed medications; hyperpharmacy was defined as use of 10 or more prescribed medications.

### QT-Prolonging Medication Prescription Characterization

Within each of the 7 QT-prolonging medication new-user cohorts, we characterized the timing of the newly prescribed QT-prolonging medication relative to acute care encounters and identified the type of clinician who prescribed the medication and the type of pharmacy that dispensed the medication. First, we examined whether the study medications were newly prescribed after acute care encounters, including urgent care or emergency department visits or hospitalizations. Eligible urgent care visits were those occurring within 1 week before the QT-prolonging medication new use. Eligible emergency department visits were those occurring within 1 week before the QT-prolonging medication new use when the QT-prolonging medication was prescribed by an emergency department clinician. Eligible hospitalizations were inpatient hospital stays with discharge dates falling within 1 week before the QT-prolonging medication new use.

Second, we used the prescriber and pharmacy characteristics in Medicare Part D claims files to characterize the prescriber and dispensing pharmacy of the QT-prolonging medications. Prescriber credentials and specialty type were ascertained from clinician specialty and credentials reported in the prescriber characteristics file and defined according to the National Uniform Claim Committee’s Health Care Provider Taxonomy codes.^26^ Pharmacies were categorized as either commercial or retail, institutional (eg, hospital or skilled nursing facilities), mail order, or other based on data in the Centers for Medicare & Medicaid Services pharmacy characteristics file.^27^

Third, we characterized the concomitant use of selected medications known to interact with the 7 most frequently filled QT-prolonging medications with known TdP risk. We used Medicare Part D claims to ascertain each beneficiary’s outpatient use of a potentially interacting medication of interest on the date of the new-use prescription fill for the study medication. Pharmacodynamically interacting drugs of interest included other QT-prolonging medications with a known risk of TdP (eTable 2 in Supplement 1). Drugs known to inhibit the metabolism of the new-use study medications (ie, cytochrome P450 [CYP] 3A4 and CYP2C19 isoenzyme inhibitors) were considered as potential pharmacokinetic interactors (eTable 4 in Supplement 1). We used the University of Washington Drug Interaction Database^28^ to compile the list of relevant CYP inhibitors.

### Statistical Analysis

We described baseline characteristics of the cohort of Medicare FFS beneficiaries receiving outpatient hemodialysis by their use (vs nonuse) status of any QT-prolonging medication with known TdP risk in 2019. Within the 7 new-user cohorts, we reported the numbers and frequencies of new-use episodes of study drugs relative to the presence (vs the absence) of acute care events as well as the prescribers and dispensing pharmacies for these new-use episodes. To assess the prevalence of concomitant use of medications that may have potentially interacted with a newly prescribed QT-prolonging medication with known TdP risk, we described the number and percentage of new-use episodes of known TdP risk medications during which patients were taking a medication with potential to pharmacodynamically and, separately, pharmacokinetically interact with the new-use medication on the date of the new-use fill. For each interacting drug pair, we described the frequency of pairs prescribed by different clinicians or the same clinician (by specialty) and those dispensed by different pharmacies or the same pharmacy (by type). All statistical analyses were performed from October 20, 2022, to June 16, 2023, using SAS, version 9.4 (SAS Institute Inc).

## Results

### Overall Study Population

Of the 20 761 individuals receiving in-center maintenance hemodialysis on January 1, 2019 (mean [SD] age, 74 [7] years; 10 150 [48.9%] female and 10 611 [51.1%] male), 10 992 (52.9%) filled an outpatient prescription for a QT-prolonging medication with known TdP risk in 2019 (eFigure in Supplement 1). Table 1 displays baseline characteristics of the cohort. Among the 10 992 users of medication with known TdP risk in the cohort, the mean (SD) age was 74 (7) years; 56.2% had a low-income subsidy; and 28.0% were Black, 6.4% were Hispanic, 56.0% were non-Hispanic White, and 9.7% were of other race or ethnicity. Compared with nonusers, users of QT-prolonging medications with known TdP risk were more frequently female (52.6% vs 44.7%) and non-Hispanic White (56.0% vs 47.9%) and had higher rates of heart failure (53.1% vs 44.2%), arrhythmias (42.4% vs 33.2%), depression (26.8% vs 16.1%), polypharmacy (65.0% vs 49.6%), and hyperpharmacy (17.7% vs 8.8%).

**Table 1.** Baseline Patient Characteristics by Use and Nonuse of QT-Prolonging Medications With Known TdP Risk in 2019^a^

Characteristic	Patients, No. (%)		
	Overall (N = 20 761)	Use of medication with known TdP risk (n = 10 992)^b^	Nonuse of medication with known TdP risk (n = 9769)^b^
Demographic and clinical characteristics			
Age, mean (SD), y^c^	74 (7)	74 (7)	74 (7)
Sex			
Female	10 150 (48.9)	5784 (52.6)	4366 (44.7)
Male	10 611 (51.1)	5208 (47.4)	5403 (55.3)
Race and ethnicity			
Black	6459 (31.1)	3073 (28.0)	3386 (34.7)
Hispanic	1300 (6.3)	699 (6.4)	601 (6.2)
Non-Hispanic White	10 834 (52.2)	6155 (56.0)	4679 (47.9)
Other^d^	2168 (10.4)	1065 (9.7)	1103 (11.3)
Social factors			
Low-income subsidy	11 305 (54.5)	6173 (56.2)	5132 (52.5)
Alcohol or drug use or dependence	669 (3.2)	378 (3.4)	291 (3.0)
Tobacco use	1690 (8.1)	956 (8.7)	734 (7.5)
History of noncompliance	1738 (8.4)	981 (8.9)	757 (7.7)
Selected comorbid conditions			
Diabetes	15 294 (73.7)	8276 (75.3)	7018 (71.8)
Ischemic heart disease	10 891 (52.5)	6193 (56.3)	4698 (48.1)
Heart failure	10 154 (48.9)	5839 (53.1)	4315 (44.2)
Peripheral artery disease	8053 (38.8)	4569 (41.6)	3484 (35.7)
Arrhythmia	7906 (38.1)	4664 (42.4)	3242 (33.2)
Gastroesophageal reflux disease	6627 (31.9)	4002 (36.4)	2625 (26.9)
Valvular disease	5323 (25.6)	2965 (27.0)	2358 (24.1)
Stroke	5097 (24.6)	2902 (26.4)	2195 (22.5)
Depression	4521 (21.8)	2944 (26.8)	1577 (16.1)
Anxiety	3515 (16.9)	2297 (20.9)	1218 (12.5)
Conduction disorder	3190 (15.4)	1810 (16.5)	1380 (14.1)
Cardiac defibrillator or pacemaker	2663 (12.8)	1581 (14.4)	1082 (11.1)
Liver disease	2322 (11.2)	1253 (11.4)	1069 (10.9)
Gastrointestinal bleed	1979 (9.5)	1132 (10.3)	847 (8.7)
Peptic ulcer	272 (1.3)	164 (1.5)	108 (1.1)
Health care utilization, prior 6 mo			
No. of hospitalizations^e^			
0	12 136 (58.5)	5986 (54.5)	6150 (63.0)
1	4405 (21.2)	2537 (23.1)	1868 (19.1)
≥2	4220 (20.3)	2469 (22.5)	1751 (17.9)
Use of ≥1 QT-prolonging medication according to TdP risk^b^^,^^f^			
Any	16 168 (77.9)	9545 (86.8)	6623 (67.8)
Possible	4692 (22.6)	2915 (26.5)	1777 (18.2)
Conditional	13 350 (64.3)	7755 (70.6)	5595 (57.3)
Known	8511 (41.0)	6335 (57.6)	2176 (22.3)
General outpatient medication use^f^			
Any use of prescribed medications	19 348 (93.2)	10 469 (95.2)	8879 (90.9)
Polypharmacy	11 996 (57.8)	7149 (65.0)	4847 (49.6)
Hyperpharmacy	2806 (13.5)	1945 (17.7)	861 (8.8)
Total unique prescribers, mean (SD)	2.4 (1.5)	2.5 (1.5)	2.2 (1.4)

Abbreviation: TdP, torsades de pointe.

^a^ Characteristics were ascertained in the 180-day baseline period (July 1 through December 31, 2018).

^b^ Prior use of a QT-prolonging medication with TdP risk is defined as the use of specified medications in the 180-day baseline period. CredibleMeds classifies medications that can prolong the QT interval as having a known, possible, or conditional TdP risk.^25^ Drugs with known TdP risk are those that prolong the QT interval and are clearly associated with a known risk of TdP, even when taken as recommended. Drugs with possible TdP risk are those that can cause QT prolongation but currently lack evidence for a risk of TdP when taken as recommended. Drugs with conditional TdP risk are those that are associated with TdP only under certain conditions (eg, excessive dose, in patients with conditions such as hypokalemia, or when taken with interacting drugs) or drugs that create conditions that facilitate or induce TdP (eg, cause an electrolyte disturbance that induces TdP). Lists of medications falling into each category are provided in eTable 2 in Supplement 1.

^c^ Age as of January 1, 2019.

^d^ Other race and ethnicity includes Asian or Pacific Islander, American Indian or Alaska Native, other, and unknown. This category was combined because of small individual sample sizes.

^e^ Hospitalizations within the previous 6 months (July 1 through December 31, 2018).

^f^ Use of specified medication (or medications) as of January 1, 2019. Polypharmacy (use of ≥5 prescribed medications) and hyperpharmacy (use of ≥10 prescribed medications) were not mutually exclusive.

### Use of QT-Prolonging Medications With Known TdP Risk

**Figure 1.**
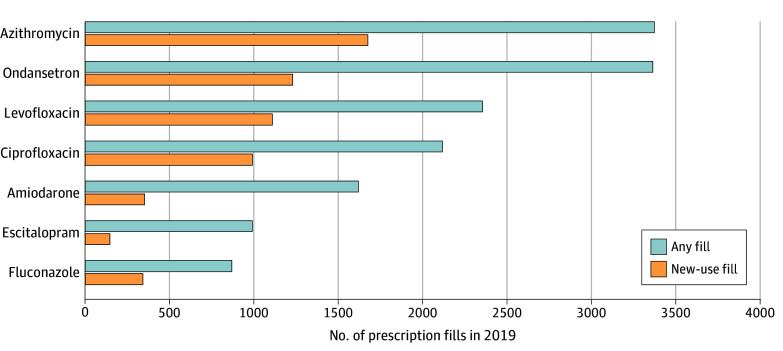
Frequently Filled QT-Prolonging Medications With Known Torsades de Pointes (TdP) Risk The 7 QT-prolonging medications with known TdP risk were the most frequently filled by Medicare fee-for-service and Part D beneficiaries receiving maintenance hemodialysis in 2019. Any fill includes repeat fills among existing users, and new-use fill indicates a prescription for the specified medication without evidence of a prior prescription for the same medication in the preceding 180 days.

Among the 10 992 Medicare beneficiaries with 1 or more prescription fills of a medication with QT-prolonging potential in 2019, 57.6% were prescribed medications with known TdP risk, and 86.8% were prescribed medications with any (ie, known, possible, or conditional) TdP risk during baseline (Table 1). During 2019, users of QT-prolonging medications with known TdP risk had a mean (SD) of 5.5 (6.1) fills of such medications and 14.8 (15.5) fills of medications with any TdP risk (eTable 5 in Supplement 1). The majority of people receiving known TdP risk medications were prescribed antibacterials or antifungals (azithromycin [30.7%], levofloxacin [21.4%], ciprofloxacin [19.3%], fluconazole [7.9%], ondansetron [30.6%], amiodarone [14.7%], or escitalopram [9.0%]) (eTable 3 in Supplement 1). As shown in Figure 1, these medications were among the most frequently filled overall (including repeat fills among existing users) and also the most common QT-prolonging medications with new-use episodes.

**Figure 2.**
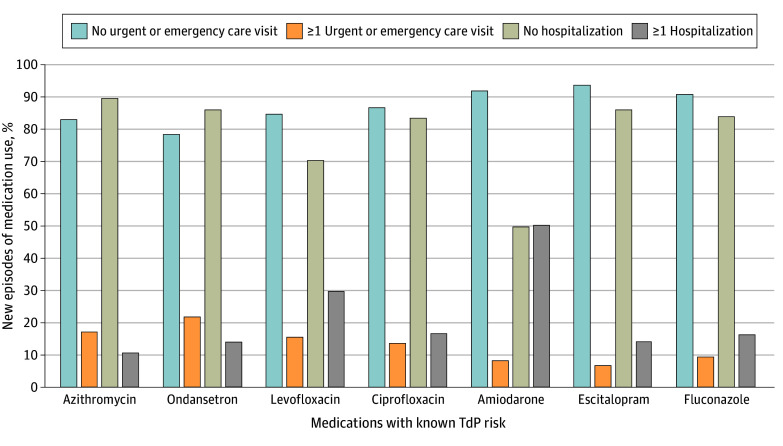
Frequency of Acute Care Events Preceding New Use of QT-Prolonging Medications With Known Torsades de Pointes (TdP) Risk by Medication Timing of medication fills, relative to acute care events, of the 7 QT-prolonging medications with known TdP risk most frequently filled by Medicare fee-for-service and Part D beneficiaries receiving maintenance hemodialysis in 2019. Acute care events were captured using Medicare Parts A and B claims for urgent care visits, emergency department visits, and hospitalizations (including observation stays). Hospitalizations and urgent care or emergency department visits were not mutually exclusive.

Approximately 80% (from 78.6% for odansetron to 93.9% for escitalopram) of new-use episodes of any QT-prolonging medication with known TdP risk occurred outside temporal proximity to an acute care event. Fewer than one-quarter (from 6.1% for escitalopram to 21.4% for odansetron) of new-use episodes of the study medications occurred within 1 week of an urgent care or emergency department visit, and for new-use episodes occurring within 1 week of a hospitalization, percentages ranged from 10.3% (for azithromycin) to 50.2% (for amiodarone) (Figure 2).

### Prescriber and Pharmacy Characteristics of QT-Prolonging Medications With Known TdP Risk

Users of QT-prolonging medication had a mean (SD) number of different prescribers of outpatient medications of 2.5 (1.5) in use on January 1, 2019 (Table 1). Of new-use prescriptions for the study QT-prolonging medications with known TdP risk during 2019, most (80.2%) came from nonnephrologists (Table 2). Whereas 10.5% to 19.8% of prescriptions for QT-prolonging medications with known TdP risk originated from nephrologists, 36.8% to 61.0% of such prescriptions originated from general medicine clinicians. Over three-quarters (75.3%) of all new-use prescriptions for QT-prolonging medications with known TdP risk in 2019 were ordered by medical doctors or doctors of osteopathy, followed by advanced practice clinicians (13.6%-19.6% of new prescriptions written by advanced practice clinicians). Most new-use prescriptions for QT-prolonging medications with known TdP risk (across the 7 new-user cohorts) were dispensed by commercial retail pharmacies (72.3%-90.1%).

**Table 2.** Origin of Prescriptions for New Use of QT-Prolonging Medications With Known TdP Risk in 2019 by Medication^a^

Prescription origin	Medication, No. (%)						
	Azithromycin	Ondansetron	Levofloxacin	Ciprofloxacin	Amiodarone	Escitalopram	Fluconazole
Medication use, No.							
New-use episodes	3168	2546	2203	1875	638	310	682
First new-use episodes	3018	2463	2130	1816	634	310	665
First new-use episodes with no hospitalization 1 wk prior to fill	2708	2123	1501	1518	316	267	559
Pharmacy characteristics							
Commercial or retail	2439 (90.1)	1588 (74.8)	1224 (81.5)	1324 (87.2)	250 (79.1)	193 (72.3)	456 (81.6)
Hospital or institutional	263 (9.7)	519 (24.4)	275 (18.3)	190 (12.5)	64 (20.3)	70 (26.2)	102 (18.2)
Mail order	SC	SC	SC	SC	SC	SC	SC
Other	SC	SC	SC	SC	SC	SC	SC
Prescriber specialty							
Emergency medicine	196 (7.2)	235 (11.1)	96 (6.4)	91 (6.0)	SC	SC	13 (2.3)
Medicine	1979 (73.1)	1443 (68.0)	1033 (68.8)	944 (62.2)	251 (79.4)	210 (78.7)	405 (72.5)
Cardiology	NR	NR	SC	SC	99 (31.3)	SC	SC
Infectious diseases	SC	SC	NR	NR	SC	SC	SC
Nephrology	536 (19.8)	242 (11.4)	201 (13.4)	249 (16.4)	NR	28 (10.5)	67 (12.0)
General medicine	1152 (42.5)	1001 (47.2)	698 (46.5)	559 (36.8)	120 (38.0)	163 (61.0)	271 (48.5)
Other medicine^b^	261 (9.6)	186 (8.8)	114 (7.6)	111 (7.3)	20 (6.3)	18 (6.7)	60 (10.7)
Hospitalist	16 (0.6)	20 (0.9)	23 (1.5)	11 (0.7)	SC	SC	SC
Obstetrics and gynecology	SC	SC	SC	SC	SC	SC	18 (3.2)
Other specialty^c^	37 (1.4)	16 (0.8)	63 (4.2)	81 (5.3)	SC	SC	17 (3.0)
Pain, palliative care, and anesthesia	SC	SC	SC	SC	SC	SC	SC
Psychiatry	SC	SC	SC	SC	SC	13 (4.9)	SC
Surgery	16 (0.6)	56 (2.6)	71 (4.7)	152 (10.0)	SC	SC	15 (2.7)
Unknown	457 (16.9)	345 (16.3)	210 (14.0)	234 (15.4)	49 (15.5)	40 (15.0)	85 (15.2)
Prescriber credentials							
Physician^d^	2046 (75.6)	1607 (75.7)	1202 (80.1)	1208 (79.6)	259 (82.0)	212 (79.4)	421 (75.3)
Advanced practice clinician^e^	530 (19.6)	402 (18.9)	225 (15.0)	240 (15.8)	43 (13.6)	41 (15.4)	109 (19.5)
Anesthesia	SC	SC	SC	SC	SC	SC	SC
Midwife or registered nurse	34 (1.3)	15 (0.7)	SC	12 (0.8)	SC	SC	SC
Unknown	95 (3.5)	96 (4.5)	66 (4.4)	57 (3.8)	13 (4.1)	12 (4.5)	18 (3.2)

Abbreviations: NR, nonreported number; SC, small cell size; TdP, torsades de pointe.

^a^ The table reports SC (n = <11) and selected cell sizes (NR; 11 to 30) for data that may be identifiable, according to the Centers for Medicare & Medicaid Services’ data-use reporting requirements.

^b^ Includes medical genetics and allergy and immunology.

^c^ Includes podiatry, physical medicine and rehabilitation, otolaryngology, dentistry, dermatology, radiology, legal medicine, neuromusculoskeletal medicine, nuclear medicine, optometry, and pathology.

^d^ Includes medical doctor and doctor of osteopathy.

^e^ Includes nurse practitioner and physician assistant.

### Prescriptions for Medications With Potential for Drug-Drug Interactions

Between 6.1% and 37.1% of new-use prescriptions for QT-prolonging medications with known TdP risk were filled in the setting of an existing prescription for a potentially interacting medication (Table 3 and eTable 6 in Supplement 1). For example, between 16.4% and 26.2% of new QT-prolonging medication prescriptions with known TdP risk occurred with the use of another pharmacodynamically interacting QT-prolonging medication. Of new ondansetron prescriptions, 23.6% occurred when patients had an existing prescription for a drug with pharmacodynamic-interaction potential (ie, another medication with known TdP risk) on the date of ondansetron new use (Table 3), and 37.1% of new escitalopram prescriptions occurred when patients were taking a study CYP2C19 inhibitor (ie, a drug with potential pharmacokinetic-interaction risk) on the date of escitalopram new use (eTable 6 in Supplement 1).

**Table 3.** Practitioner Characteristics of Common Pharmacodynamically Interacting Medication Pairs Among New Users of QT-Prolonging Medications With Known TdP Risk in 2019^a^

Characteristic	Medication, No. (%)						
	Azithromycin	Ondansetron	Levofloxacin	Ciprofloxacin	Amiodarone	Escitalopram	Fluconazole
New-use episodes, No.	3168	2546	2203	1875	638	310	682
First new episodes, No.	3018	2463	2130	1816	634	310	665
Concurrent use with another QT-prolonging medication with known TdP risk^b^	516 (17.1)	582 (23.6)	463 (21.7)	367 (20.2)	104 (16.4)	54 (17.4)	174 (26.2)
Pharmacies							
Different	83 (16.1)	64 (11.0)	68 (14.7)	48 (13.1)	15 (14.4)	SC	16 (9.2)
Same	433 (83.9)	518 (89.0)	395 (85.3)	319 (86.9)	89 (85.6)	51 (94.4)	158 (90.8)
Commercial or retail	363 (83.8)	359 (69.3)	298 (75.4)	268 (84.0)	67 (75.3)	31 (60.8)	125 (79.1)
Institutional^c^	69 (15.9)	153 (29.5)	93 (23.5)	47 (14.7)	20 (22.5)	19 (37.3)	30 (19.0)
Other	SC	SC	SC	SC	SC	SC	SC
Mail order	SC	SC	SC	SC	SC	SC	SC
Prescribers							
Different	338 (65.5)	315 (54.1)	292 (63.1)	234 (63.8)	64 (61.5)	25 (46.3)	96 (55.2)
Same	178 (34.5)	267 (45.9)	171 (36.9)	133 (36.2)	40 (38.5)	29 (53.7)	78 (44.8)
General medicine^d^	121 (68.0)	195 (73.0)	130 (76.0)	82 (61.7)	30 (75.0)	25 (86.2)	51 (65.4)
Nephrology	15 (8.4)	11 (4.1)	11 (6.4)	16 (12.0)	SC	SC	SC
Other^e^	26 (14.6)	36 (13.5)	17 (9.9)	20 (15.0)	SC	SC	13 (16.7)
Unknown	16 (9.0)	25 (9.4)	13 (7.6)	15 (11.3)	SC	SC	SC

Abbreviations: SC, small cell size; TdP, torsades de pointes.

^a^ The table reports SC (<11) for data that may be identifiable, according to the Centers for Medicare & Medicaid Services’ data-use reporting requirements.

^b^ Denominators for pharmacy and prescriber data.

^c^ Pharmacy within long-term care facility.

^d^ Includes internal medicine, family medicine, and general practice.

^e^ Includes emergency medicine, cardiology, infectious diseases, hospitalist medicine, psychiatry, surgery, medical genetics, allergy, immunology, podiatry, physical medicine and rehabilitation, otolaryngology, dentistry, dermatology, radiology, legal medicine, neuromusculoskeletal medicine, nuclear medicine, optometry, and pathology.

We observed different prescribing patterns across pharmacodynamically and pharmacokinetically interacting medications. Roughly half of new prescriptions for study QT-prolonging medications occurring in the setting of concomitant use of another QT-prolonging medication with known TdP risk were written by different prescribers (46.3%-65.5%), and most were dispensed by the same pharmacies (83.9%-94.4%) (Table 3 and eTable 6 in Supplement 1). When 2 drugs with potential for pharmacodynamic interaction were dispensed by the same pharmacy, the dispensing pharmacy was most often a commercial or retail pharmacy. When 2 drugs with potential for pharmacodynamic interaction were prescribed by the same clinician, the clinician was most often a nonnephrologist. In contrast, more than half (53.9%) of new-use escitalopram prescriptions in the setting of concurrent use of a study CYP2C19 inhibitor (drugs with potential for pharmacokinetic interaction) were dispensed by the same commercial or retail pharmacy (89.8%) and written by different prescribers (53.3%) (eTable 6 in Supplement 1).

## Discussion

In this cross-sectional study, the findings suggest that Medicare beneficiaries aged 60 years or older who have hemodialysis-dependent kidney failure were commonly prescribed QT-prolonging medications with known TdP risk by nonnephrology clinicians and that such prescriptions often originated from nonacute care settings. The findings also suggest that up to one-third of new-use prescriptions for the most commonly prescribed QT-prolonging medications were filled in the setting of concomitant use of a potentially interacting medication and that prescriptions for the medications with potential for interaction often originated from different prescribers.

Existing data demonstrate that individuals with dialysis-dependent kidney failure are prescribed QT-prolonging medications at higher rates than people without kidney failure and that patients receiving dialysis who have risk factors for drug-induced QT prolongation (ie, female sex, advanced age, comorbid heart failure, and concurrent use of ≥1 QT-prolonging medication) are exposed to medications with known TdP risk more often than individuals without such risk factors.^29^ Moreover, comparative safety studies of some QT-prolonging medications with known TdP risk (eg, citalopram, escitalopram, and levofloxacin) show that, compared with use of clinically relevant comparator medications, use of QT-prolonging medications with known TdP risk is associated with a higher risk of sudden cardiac death, a potential drug-related complication.^12,14^ Furthermore, prior studies have shown elevated sudden cardiac death risk when patients are prescribed QT-prolonging drugs in the setting of concomitant use of drugs with potential for pharmacodynamic and pharmacokinetic interaction.^12,30^ Together with our findings, these data underscore the importance of identifying strategies to mitigate unsafe medication-prescribing practices for people receiving hemodialysis.

To our knowledge, there is no published research on the origins of prescriptions for people receiving maintenance hemodialysis. Our data provide new insights on the origins and contexts of high-risk medications among patients receiving hemodialysis. We found that less than one-quarter of prescriptions for QT-prolonging medications with known TdP risk occurred within 1 week of an acute care encounter. Instead, the majority of these medications were prescribed from nonhospital settings, suggesting that hospital-based medication-monitoring systems, on their own, may be inadequate to mitigate harm from unsafe medication-prescribing practices.

We focused our analysis on medications with known TdP risk because we sought to examine medications relevant to sudden cardiac death, the leading cause of death in the population undergoing hemodialysis. Identifying potentially harmful prescription patterns is essential, as medication use is a potentially modifiable risk factor for sudden cardiac death, unlike risk factors more challenging to modify (eg, change in ventricular architecture, fluid and electrolyte shifts inherent to dialysis procedures). However, it is likely that our study’s findings are a microcosm of a larger issue of risky prescription practices among individuals receiving maintenance hemodialysis. Investigation of the prescription and dispensation patterns of other potentially harmful medications (eg, opioids,^31,32^ benzodiazepines,^32^ sedative hypnotics,^33,34^ and muscle relaxants^35^) is warranted.

Our findings highlight opportunities for the uptake of strategies targeting outpatient prescribers and dispensing pharmacies to reduce drug-related harms of high-risk medications for clinically complex patients. In the population undergoing dialysis, educating nonnephrology clinicians about medication safety concepts specific to kidney failure is one potential approach to reducing medication-related complications.^36^ However, this approach is unlikely to be successful on its own due to the diversity of clinicians contributing to the care of patients receiving dialysis.^37,38,39^ Other strategies shown to be effective in the outpatient setting include pharmacist-led medication reconciliation and medication management programs^40,41,42,43,44^ as well as interoperable EHRs and clinical decision support systems.^36,37,45,46^ Pharmacist-led medication reconciliation, the process of generating an accurate and complete list of patient medications, inclusive of prescription and over-the-counter medications, herbals, and supplements,^44^ can be effective for reducing medication-related harm.^40,41,42,43^ The process falls short when clinicians lack up-to-date information on patient comorbid conditions and medication use. Gaps in information are particularly common in dialysis care because the majority of US dialysis clinics use EHR systems that do not communicate with local health system EHRs.^36,47^ Such challenges with interoperable EHRs and clinical decision support systems^45,46^ and missteps in cross-clinician communication have been associated with reliance on the dialysis clinic for detection of potentially dangerous prescribing practices.^37^

In 2022, the Centers for Medicare & Medicaid Services introduced a medication reconciliation measure to the End-Stage Renal Disease Quality Incentive Program, a Medicare pay-for-performance program. The safety measure requires dialysis clinics to attest to providing monthly medication reconciliation for all patients under their care. For medication reconciliation to be effective at reducing drug-related complications, it must be performed in close proximity to the unsafe prescribing event. While existing evidence supports acute care encounters as events that should trigger medication reconciliation, our finding that QT-prolonging medications with known TdP risk most often originate from nonacute ambulatory settings suggests that outpatient clinician visits should also be triggers for medication reconciliation. Areas ripe for future investigation include identification of the optimal timing, frequency, and setting for medication reconciliation as well as adoption of integrated communication systems aimed at minimizing harm from care fragmentation.^38,39^

Within the system of care, the dispensing pharmacy and prescription drug plan could play important roles in medication safety checks. We found that QT-prolonging medications with known TdP risk and their interacting medications were most commonly ordered by different prescribers but filled at the same commercial or retail pharmacy. As such, pharmacies may be well-positioned to identify potentially risky medication prescribing. Existing commercial and institutional pharmacy software systems detect prescriptions for contraindicated and/or incorrectly dosed medications as well as medication combinations with potential for interaction. However, these systems are effective only to the extent that pharmacy-ordering systems can cross-check for interacting medications at the time of prescription fill and that this cross-check occurs within and across affiliated pharmacy sites (ie, chain retail pharmacies), when the data underlying the alerts are accurate, and when dispensing pharmacists act on the triggered alerts. Pharmacy systems, especially in community settings, may not be connected to EHR systems and thus lack up-to-date clinical information.^36,48^ However, prescription drug plans may be better positioned in this regard, as they collect prescription drug information at the point of pharmacy purchase. To maximize the potential effectiveness of medication-monitoring systems and reconciliation among individuals with dialysis-dependent kidney failure, as well as patients more broadly, information systems that integrate data from care settings, dispensing pharmacies, and prescription drug plans are needed.

### Limitations

Our findings should be considered in the context of study limitations. First, we described prescription medication fills, which may not have reflected patients’ actual medication use. Similarly, we were unable to account for prescription adjustments initiated by clinicians in response to a perceived change in risk-benefit that might have occurred after prescription fill (ie, verbally communicated and not documented). Second, our data source captured medications obtained under Medicare prescription drug plans and did not include medications purchased without insurance, under other insurance plans (eg, Medicare Advantage in 2019,^49^ private insurance), or over the counter. The validity of claims’ file prescriber and pharmacy data was unknown; misclassification of clinician specialty, credentials, or pharmacy characteristics was possible. Last, our analyses were descriptive; we did not investigate potential associations of prescription origins with risks of adverse clinical outcomes.

## Conclusions

In this cross-sectional study, Medicare beneficiaries with kidney failure receiving hemodialysis were commonly prescribed QT-prolonging medications with known TdP risk maintenance by nonnephrology clinicians and from nonacute settings. Prescriptions for potentially interacting medications often originated from different prescribers. These findings highlight the need to better coordinate medication management at the clinician and health system levels and suggest that both prescriber- and pharmacy-targeted strategies may be important to minimize drug-related complications in the high-risk population undergoing dialysis.

## Notes

Supplement 1. **eFigure.** Cohort Sampling Scheme **eTable 1.** Data Sources: Diagnosis Codes, Data Files for Social Factors, Comorbid Conditions, and Health Care Utilization **eTable 2.** List of QT-Prolonging Medications With a Known, Possible, and Conditional Risk of TdP **eTable 3.** Most Frequent QT-Prolonging Medications With Known TdP Risk Filled in 2019 **eTable 4.** Medications in CYP Inhibitor Classes **eTable 5.** QT-Prolonging Medication Use in 2019 **eTable 6.** Pharmacy and Prescriber Characteristics of the Most Common Study Pharmacokinetic Medications Interacting With New-Use Escitalopram in 2019

Supplement 2.
Data Sharing Statement
