# Supplementary material for: Prescription and Dispensation of QT-Prolonging Medications in Individuals Receiving Hemodialysis
Source: JAMA Netw Open. 2024 Apr 30;7(4):e248732. doi: 10.1001/jamanetworkopen.2024.8732 (PMC11061769; doi:10.1001/jamanetworkopen.2024.8732)
Supplement: Supplement 1. — eFigure. Cohort Sampling Scheme eTable 1. Data Sources: Diagnosis Codes, Data Files for Social Factors, Comorbid Conditions, and Health Care Utilization eTable 2. List of QT-Prolonging Medications With a Known, Possible, and Conditional Risk of TdP eTable 3. Most Frequent QT-Prolonging Medications With Known TdP Risk Filled in 2019 eTable 4. Medications in CYP Inhibitor Classes eTable 5. QT-Prolonging Medication Use in 2019 eTable 6. Pharmacy and Prescriber Characteristics of the Most Common Study Pharmacokinetic Medications Interacting With New-Use Escitalopram in 2019 [file jamanetwopen-e248732-s001.pdf]

Supplemental Online Content

Wang V, Wang CH, Assimon MM, Pun PH, Winkelmayer WC, Flythe JE. Prescription and dispensation of QT-prolonging medications in individuals receiving hemodialysis. *JAMA Netw Open*. 2024;7(4):e248732. doi:10.1001/jamanetworkopen.2024.8732

eFigure. Cohort Sampling Scheme
eTable 1. Data Sources: Diagnosis Codes, Data Files for Social Factors, Comorbid Conditions, and Health Care Utilization
eTable 2. List of QT-Prolonging Medications With a Known, Possible, and Conditional Risk of TdP
eTable 3. Most Frequent QT-Prolonging Medications With Known TdP Risk Filled in 2019
eTable 4. Medications in CYP Inhibitor Classes
eTable 5. QT-Prolonging Medication Use in 2019
eTable 6. Pharmacy and Prescriber Characteristics of the Most Common Study Pharmacokinetic Medications Interacting With New-Use Escitalopram in 2019

This supplemental material has been provided by the authors to give readers additional information about their work.

© 2024 Wang V et al. *JAMA Network Open*.

eFigure. Cohort Sampling Scheme

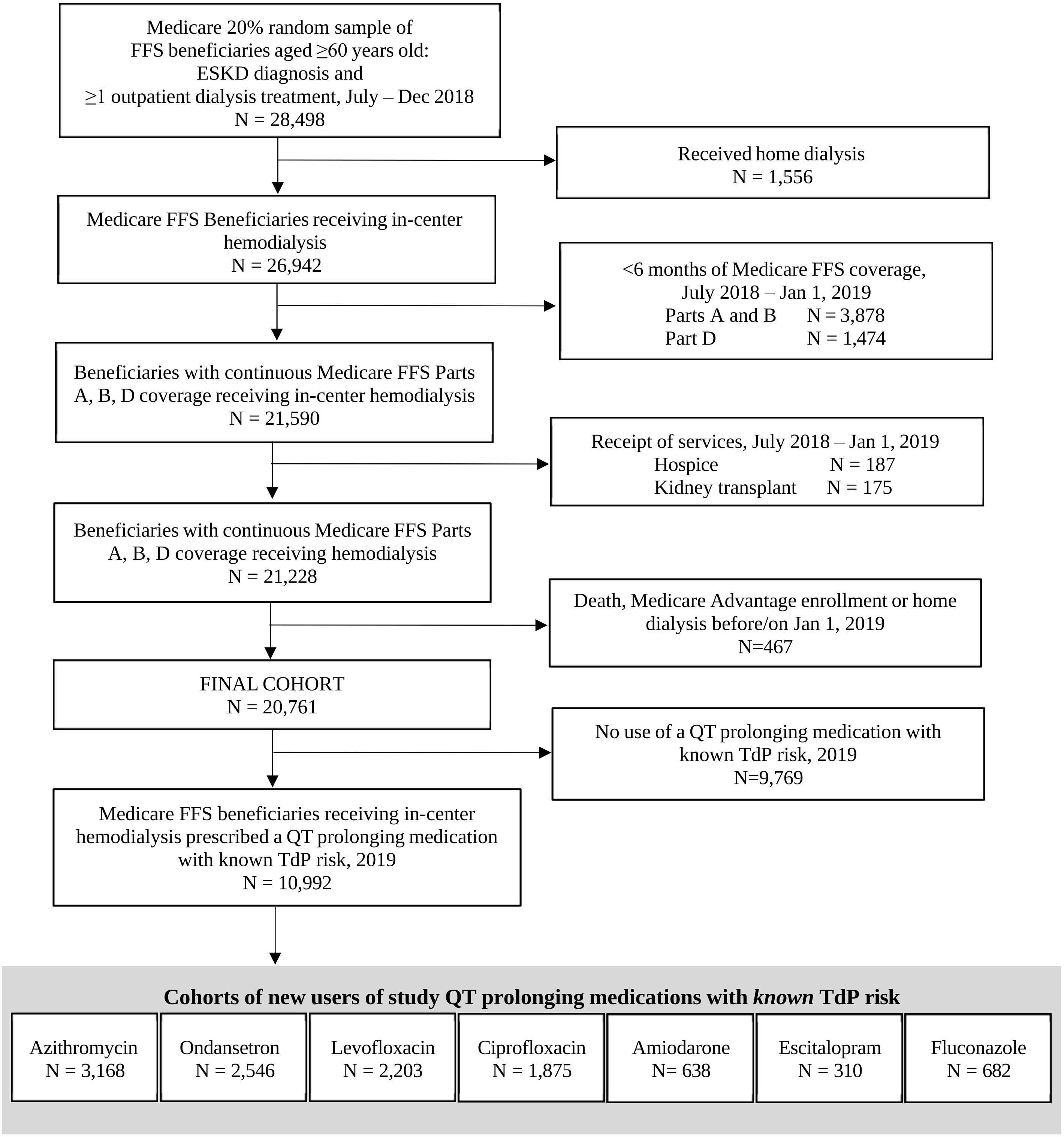

Abbreviations: ESKD, end-stage kidney disease; FFS, fee-for service; TdP, Torsades de Pointes.

© 2024 Wang V et al. *JAMA Network Open*.

eTable 1. Data Sources: Diagnosis Codes, Data Files for Social Factors, Comorbid Conditions, and Health Care Utilization

**Variable**	**Relevant ICD-10 diagnosis codes or claims data (Medicare data source)**
**Eligibility criteria**	
End-stage kidney disease	N18.5, N18.6, N19, Z91.15, Z99.2, Z94.0, I12.0, I13.11, I13.12 (Part A/B claims)
Dialysis procedure codes (including home dialysis)	CPT: 90935, 90937, 90945, 90947, 90999 (Part B claims)<br>Revenue center: 0820-0889 (Part B claims)
Kidney transplant	0TY00Z0, 0TY00Z1, 0TY00Z2, 0TY10Z0, 0TY10Z1, 0TY10Z2
**Social factors**	
Low-income subsidy	cost share group code 01-08
Alcohol or drug abuse/dependence	F10.10, F10.11, F10.120, F10.129, F10.20, F10.21, F10.220, F10.229, F11.10, F11.11, F11.120, F11.129, F11.20–F11.25, F11.28, F11.29, F11.90, F12.10, F12.11, F12.20–F12.25, F12.28, F12.29, F13.10, F13.11, F13.120, F13.20–F13.29, F13.90, F14.10, F14.11, F14.120, F14.20–F14.25, F14.28, F14.29, F14.90, F15.10, F15.11, F15.120, F15.20–F15.25, F15.28, F15.29, F15.90, F16.10, F16.11, F16.120, F16.20–F16.25, F16.28, F16.29, F16.90, F18.10, F18.11, F18.120, F18.20–F18.25, F18.27, F18.28, F18.29, F18.90, F19.10, F19.11, F19.120, F19.20–F19.29, F19.90, F55<br>(Part A/B claims)
Tobacco use	F17.200, F17.201, F17.210, F17.211, F17.220, F17.221, F17.290, F17.291 (Part A/B claims)
History of non-compliance	Z91.1 (Part A/B claims)
**Comorbid conditions**	
Arrhythmia	I46–I49 (Part A/B claims)
Conduction disorder	I44–I45 (Part A/B claims)
Dyslipidemia	E78.0, E78.1, E78.2, E78.4, E78.5 (Part A/B claims)
Heart failure	I09.81, I11.0, I13.0, I50 (Part A/B claims)
Hypertension	I10–I16 (Part A/B claims)
Ischemic heart disease	I20–I25 (Part A/B claims)
Peripheral arterial disease	E10.5, E11.5, E13.5, I70.2–I70.9, I73.1, I73.89, I73.9, I74.3–I74.5, I75.02, I77.72, I79.1, I79.8 (Part A/B claims)
Stroke	G45-G46, I60–I69 (Part A/B claims)
Valvular disease	I05–I08, I09.1, I34–I37 (Part A/B claims)
Implantable cardioverter defibrillator	Z95.810 (Part A/B claims)
Cardiac pacemaker	Z95.0 (Part A/B claims)
Anxiety	F41, F43.22, F43.23, F93.0 (Part A/B claims)
Depression	F31.30–F31.32, F31.4–F31.6, F31.75–F31.78, F32.0–F32.5, F32.89, F32.9, F33.0–F33.9, F34.1, F43.21, F43.23 (Part A/B claims)
Diabetes	E10, E11, E13 (Part A/B claims)
Gastrointestinal bleed	K25.0, K25.2, K25.4, K25.6, K26.0, K26.2, K26.4, K26.6, K27.0, K27.2, K27.4, K27.6, K28.0, K28.2, K28.4, K28.6, K55.21, K57.01, K57.11, K57.13, K57.21, K57.31, K57.33, K57.41, K57.51, K57.53, K57.81, K57.91, K57.93, K62.5, K92.0, K92.1, K92.2 (Part A/B claims)
Gastroesophageal reflux disease	K21 (Part A/B claims)
Peptic ulcer	K27 (Part A/B claims)
Hypothyroidism	E00, E01.8, E02, E03.0–E03.3, E03.8, E03.9, E89.0 (Part A/B claims)
Liver disease	K70–K76 (Part A/B claims)
**Health care utilization**	
Hospitalization	Total number of inpatient hospitalizations (discharges based on the DSCHRGDT variable)<br>CLM_TYPE = 60 (Part A Claims, MEDPar files)
**Medication use**	
Any use of prescribed medications	(Part D Claims)
Number of unique prescribers	(Part D Claims)
Use of ≥ 1 medication with *known* TdP risk*	See **Table S2** for relevant medications (Part D Claims)
Use of ≥ 1 medication with *conditional* TdP risk*	See **Table S2** for relevant medications (Part D Claims)
Use of ≥ 1 medication with *possible* TdP risk*	See **Table S2** for relevant medications (Part D Claims)

Abbreviations: TdP, Torsades de Pointes.

© 2024 Wang V et al. *JAMA Network Open*.

eTable 2. List of QT-Prolonging Medications With a Known, Possible, and Conditional Risk of TdP*

**Known risk of TdP (n=40)**
Amiodarone, anagrelide, arsenic trioxide, azithromycin, cesium chloride, chloroquine, chlorpromazine, cilostazol, ciprofloxacin, citalopram, clarithromycin, cocaine, cisopyramide, dofetilide, donepezil, dronedarone, droperidol, erythromycin, escitalopram, flecainide, fluconazole, haloperidol, hydroxychloroquine, ibutilide, levofloxacin, methadone, mobocertinib, moxifloxacin, ondansetron, oxaliplatin, papaverine, pentamidine, pimozide, procainamide, propofol, quinidine, sevoflurane, sotalol, thioridazine, vandetanib
**Possible risk of TdP (n=113)**
Alfuzosin, apalutamide, apomorphine, aripiprazole, artemether/lumefantrine, asenapine, atomoxetine, bedaquiline, bendamustine, bicalutamide, bortezomib, bosutinib, buprenorphine, cabozantinib, capecitabine, ceritinib, clozapine, cobimetinib, crizotinib, dabrafenib, dasantinib, degarelix, desipramine, dextromethorphan/quinidine, dolasteron, efavirenz, eliglustat, encorafenib, entrectinib, epirubicin, eribulin, felbamate, fingolimod, fluorouracil, gemifloxacin, gilteritnib, glasdegib, granisertron, hydrocodone (ER only), iloperidone, imatinib, imipramine, inotuzumab ozogamicin, isradipine, ivosidenib, laoatinib, lefamulin, lenvatinib, leuprolide, levetiracetam, levoketoconazole, lithium, lofexidine, lopinavir/ritonavir, lumateperone, lurasidone, maprotiline, midostaurin, mifepristone, mirabegron, mirtazapine, necitumumab, nicardipine, nilotinib, nortriptyline, nusinersen, ofloxacin, oliceridine, osilordrostat, osimertinib, oxytocin, ozanimod, paliperidone, palonosetron, panobinostat, pasireotide, pazopanib, perflutren, perphenazine, pimavanserin, pitolisant, ponesimod, pretomanid, primaquine, promethazine, relugolix, remimazolam, ribociclib, rilpivirine, romidepsin, rucaparib, saquinavir, selpercantib, Siponimod, sorafenib, sunitinib, tacrolimus, tamoxifen, tazemetostat, telavancin, telithromycin, tetrabenazine, tipiracil/trifluridine, tizanidine, tolterodine, tramadol, trimipramine, valbenazine, vardenafil, vemurafenib, voclosporin, varinostat
**Conditional risk of TdP (n=48)**
Abiraterone, amantadine, amisulpride, amitriptyline, amphotericin B, atazanavir, bendroflumethiazide (also called bendrofluazide), chloralhydrate, cimetidine, clomipramine, diltiazem, diphenhydramine, doxepin, esomeprazole, famotidine, fluoxetine, fluvoxamine, furosemide, galantamine, hydrochlorothiazide, hydroxyzine, indapamide, itraconazole, ivabradine, ketoconazole, lansoprazole, loperamide, metoclopramide, metolazone, metronidazole, nelfinavir, olanzapine, omeprazole, pantoprazole, paroxetine, piperacillin/tazobactam, posaconazole, propafenone, quetiapine, quinine, ranolazine, risperidone, sertraline, solifenacin, torsemide, trazodone, voriconazole, ziprasidone

* Lists of medications were obtained from CredibleMeds. CredibleMeds classifies medications that can prolong the QT interval as having a known, possible, or conditional TdP risk. Drugs with known TdP risk are defined as drugs that prolong the QT interval *and* are clearly associated with a known risk of TdP, even when taken as recommended. Drugs with possible TdP risk are defined as drugs that can cause QT prolongation *but* currently lack evidence for a risk of TdP when taken as recommended. Drugs with conditional TdP risk are defined as drugs that are associated with TdP only under certain conditions (e.g., excessive dose, in patients with conditions such as hypokalemia, or when taken with interacting drugs) *or* drugs that create conditions that facilitate or induce TdP (e.g., cause an electrolyte disturbance that induces TdP). This list of medications was obtained from the CredibleMeds website on 6/3/2022. According to the CredibleMeds website, this specific medication list was last revised on 5/26/2022.

Abbreviations: TdP, Torsades de Pointes.

© 2024 Wang V et al. *JAMA Network Open*.

eTable 3. Most Frequent QT-Prolonging Medications With Known TdP Risk Filled in 2019

Medication	Any fill in 2019 N=10,992 patients		New fill in 2019 N=4,657 patients	
	Unique patients N (%)	Fills (N)	Unique patients N (%)	Fills (N)
**Azithromycin**	**3,372 (30.7%)**	**4,732**	**1,676 (36.0%)**	**2,161**
**Ondansetron**	**3,364 (30.6%)**	**8,569**	**1,228 (26.4%)**	**2,087**
**Levofloxacin**	**2,353 (21.4%)**	**3,358**	**1,109 (23.8%)**	**1,470**
**Ciprofloxacin**	**2,118 (19.3%)**	**3,245**	**992 (21.3%)**	**1,347**
**Amiodarone**	**1,618 (14.7%)**	**6,844**	**352 (7.6%)**	**1,047**
**Escitalopram**	**990 (9.0%)**	**5,338**	**146 (3.1%)**	**459**
**Fluconazole**	**869 (7.9%)**	**1,587**	**343 (7.4%)**	**581**
Donepezil	732 (6.7%)	4,410	134 (2.9%)	520
Citalopram	720 (6.6%)	3,944	82 (1.8%)	273
Cilostazol	228 (2.1%)	937	57 (1.2%)	161
Clarithromycin	97 (0.9%)	103	56 (1.2%)	59
Hydroxychloroquine	93 (0.8%)	477	SC	26
Haloperidol	85 (0.8%)	306	21 (0.5%)	30
Dronedarone	60 (0.5%)	338	10 (0.2%)	29
Moxifloxacin	55 (0.5%)	71	32 (0.7%)	43
Methadone	50 (0.5%)	362	10 (0.2%)	39
Chlorpromazine	42 (0.4%)	155	11 (0.2%)	24
Sotalol	28 (0.3%)	138	SC	44
Erythromycin	24 (0.2%)	64	SC	SC
Flecainide	23 (0.2%)	89	SC	13

Abbreviations: TdP, Torsades de Pointes.

© 2024 Wang V et al. *JAMA Network Open*.

eTable 4. Medications in CYP Inhibitor Classes

Class	Medications
CYP3A4 inhibitors	amprenavir, aprepitant, atazanavir, atazanavir/ ritonavir, berotralstat, casopitant, cimetidine, ciprofloxacin, crizotinib, darunavir, darunavir/ritonavir, diltiazem, dronedarone, duvelisib, erythromycin, faldaprevir, fedratinib, fluconazole, imatinib, ipatasertib, isavuconazole, istradefylline, lefamulin, letermovir, netupitant, nilotinib, ravuconazole, schisandra sphenanthera, tofisopam, verapamil, voxelotor
CYP2C19 inhibitors	Ticlopidine, cannabidiol, efavirenz, etravirine, fedratinib, fexinidazole, moclobemide, stiripentol, triclabendazole, voriconazole, omeprazole, esomeprazole, pantoprazole, lansoprazole, dexlansoprazole, and rabeprazole

Abbreviations: CYP, cytochrome p450.

© 2024 Wang V et al. *JAMA Network Open*.

eTable 5. QT-Prolonging Medication Use in 2019

	Overall	Existing users of QT prolonging medications with *known* TdP risk	New users of QT prolonging medications with *known* TdP risk
N	10,992	6,335 (57.6%)	4,657 (42.4%)
QT prolonging medication fills in 2019, mean ± SD; median [Q1-Q3]			
Any TdP risk	12.7 ± 14.0<br>9 [4-16]	14.8 ± 15.5<br>11 [6-19]	9.7 ± 11.0<br>7 [3-13]
Known TdP risk	4.1 ± 5.1<br>2 [1-5]	5.5 ± 6.1<br>4 [2-7]	2.2 ± 2.1<br>1 [1-3]
Conditional TdP risk	6.7 ± 9.4<br>4 [1-9]	7.3 ± 10.0<br>5 [1-10]	5.9 ± 8.5<br>4 [0-8]
Possible TdP risk	1.8 ± 4.6<br>0 [0-1]	2.0 ± 5.0<br>0 [0-2]	1.5 ± 4.1<br>0 [0-1]
Types of QT prolonging medications filled in 2019, mean ± SD; median [Q1-Q3]			
Any TdP risk	3.5 ± 1.8<br>3 [2-5]	3.7 ± 1.9<br>3 [2-5]	3.1 ± 1.7<br>3 [2-4]
Known TdP risk	1.5 ± 0.8<br>1 [1-2]	1.7 ± 0.9<br>1 [1-2]	1.3 ± 0.6<br>1 [1-2]
Conditional TdP risk	1.5 ± 1.2<br>1 [1-2]	1.6 ± 1.3<br>1 [1-2]	1.4 ± 1.2<br>1 [0-2]
Possible TdP risk	0.4 ± 0.6<br>0 [0-1]	0.5 ± 0.7<br>0 [0-1]	0.4 ± 0.6<br>0 [0-1]

Abbreviations: SD, standard deviation; Q, quartile; TdP, Torsades de Pointes.

© 2024 Wang V et al. *JAMA Network Open*.

eTable 6. Pharmacy and Prescriber Characteristics of the Most Common Study Pharmacokinetic Medications Interacting With New-Use Escitalopram in 2019[a]

	Escitalopram new-use (N=310)	
	**CYP2C19 inhibitors**	**CYP3A4 inhibitors**
**Concurrent use, N (%)**	**115 (37.1%)**	**19 (6.1%)**
**Different Pharmacies, N (%)**	17 (20.1%)	3 (15.8%)
**Same Pharmacy, N (%)**	150 (89.8%)	16 (84.2%)
Commercial/ Retail	112 (67.1%)	14 (73.7%)
Institutional (Long-term care facility)	36 (21.6%)	SC
Other	SC	SC
Mail order	SC	SC
**Different Prescribers, N (%)**	89 (53.3%)	15 (78.9%)
**Same Prescriber, N (%)**	78 (46.7%)	4 (21.1%)
General Medicine[b]	56 (33.5%)	SC
Nephrology	SC	SC
Other[b]	NR	SC

Note: Bolded sample (N) describing new-use of escitalopram and concurrent use of select CYP inhibitors serves as the denominator for the pharmacy and prescriber statistics below. The table reports small cell sizes <11 and selected cell sizes n=11-30 ("non-reported") for data that may be identifiable, as per CMS data use reporting requirements.

[a] Lists of medications falling into the CYP2C19 and CYP3A4 inhibitor categories are provided in **eTable 4**. Escitalopram was selected as the exemplar new-use QT-prolonging medication because of its status as a major substrate of CYP2C19 and CYP3A4, for which concurrent use of a CYP2C19 or CYP3A4 inhibitor(s) could inhibit escitalopram's metabolism and result in accumulation and an increased risk of adverse events.

[b] General Medicine includes Internal Medicine, Family Medicine, and General Practice. Other prescribers include Emergency Medicine, Cardiology, Infectious Diseases, Hospitalist Medicine, Psychiatry, Surgery, Medical Genetics, Allergy, Immunology, Podiatry, Physical Medicine and Rehabilitation, Otolaryngology, Dentistry, Dermatology, Radiology, Legal Medicine, Neuromusculoskeletal Medicine, Nuclear Medicine, Optometry, and Pathology.

Abbreviations: CMS, Centers for Medicare and Medicaid Services; CYP, cytochrome p450; NR, non-reported; SC: small cell size.

© 2024 Wang V et al. *JAMA Network Open*.
