# Supplementary material for: Prescription and Dispensation of QT-Prolonging Medications in Individuals Receiving Hemodialysis
Source: JAMA Netw Open. 2024 Apr 30;7(4):e248732. doi: 10.1001/jamanetworkopen.2024.8732 (PMC11061769; doi:10.1001/jamanetworkopen.2024.8732)
Supplement: Supplement 2. — Data Sharing Statement [file jamanetwopen-e248732-s002.pdf]

Data Sharing Statement

Wang. Prescription and Dispensation of QT-Prolonging Medications in Individuals Receiving Hemodialysis. *JAMA Netw Open*. Published April 29, 2024.
doi:10.1001/jamanetworkopen.2024.8732

Data
Data available: No

Additional Information
Explanation for why data not available: The data used in this study are publicly available from CMS with the execution of a data use agreement.
